# Experiences of Formal and Informal Support Among Adult-Daughter Caregivers of People With Dementia in Sweden: A Qualitative Study

**DOI:** 10.1177/14713012251376774

**Published:** 2025-09-08

**Authors:** Oscar Blomberg, Abla Sami, Paul Farrand, Renita Sörensdotter, Frida Svedin, Anders Brantnell, Louise von Essen, Anna Cristina Åberg, Joanne Woodford

**Affiliations:** 1 CIRCLE – Complex Intervention Research in Health and Care, Department of Women´s and Children´s Health, 8097 Uppsala University, Uppsala, Sweden; 2 Clinical Education, Development and Research (CEDAR), Psychology, 3286 University of Exeter, Devon, UK; 3 Centre for Gender Research, 8097 Uppsala University, Uppsala, Sweden; 4 Division of Industrial Engineering and Management, Department of Civil and Industrial Engineering, 8097 Uppsala University, Uppsala, Sweden; 5 Medical Science, School of Health and Welfare, 101092 Dalarna University, Falun, Sweden; 6 Department of Public Health and Caring Sciences, Clinical Geriatrics, 8097 Uppsala University, Uppsala, Sweden

**Keywords:** adult-daughter, caregiver, dementia, support, formal, informal

## Abstract

People with dementia are living longer in the community and reliance on informal caregivers is increasing. Few studies have focused on the experiences of adult-daughter caregivers (daughter caregivers), who may have increased risk of depression and lack of support compared with spousal caregivers. We aimed to explore the experiences of accessing and receiving formal and informal support among daughter caregivers of people living with dementia in Sweden. We conducted semi-structured interviews with 23 daughter caregivers. A topic guide was used to explore experiences of accessing and receiving formal and informal support in their caregiving role. We analyzed the interviews using reflexive thematic analysis. We generated two themes: Types of support, and Mismatched support. The first theme, with subthemes *Sharing experiences, Professional support*, and *Getting some relief*, captured the caregiver support context with different types of support accessed and received by daughter caregivers, with peer support and informational support identified as important components of caregiver support. The second theme, with subthemes *Lack of tailoring to daughter caregivers’ needs* and *Difficulties navigating support*, captured how daughter caregivers perceived existing support to be inadequate to meet their specific needs and the complex structure of available support limited their opportunities to access support. Daughter caregivers experience a mismatch between the type of support accessed and received and their specific support needs. Support better tailored to the needs and situation of daughter caregivers of people with dementia in Sweden is required. The potential of utilizing peer support for caregivers warrants further research.

## Introduction

Approximately 57 million people are living with dementia worldwide (GBD 2019 Dementia Forecasting Collaborators, 2022) and, with rising life expectancy, a growing number of people with dementia are living longer in the community (Meinow et al., 2022; Sleeman et al., 2019). People living with dementia have higher risk of depression (Leung et al., 2021), poor quality of life (Burks et al., 2021), and increased mortality (Taudorf et al., 2021). As a progressive and neurodegenerative syndrome, the need for care and support increases as the dementia advances (Nguyen et al., 2020).

In Sweden, the majority of the estimated 150,000 people living with dementia reside in the community rather than residential care (Sm-Rahman et al., 2021). However, health and social care policies aiming to support older people to live in the community for longer, alongside concomitant reductions in formal health and social care provision, have led to an increasing reliance on informal caregivers (caregivers; Dahlberg et al., 2018; Ulmanen & Szebehely, 2015). Caregivers are commonly partners/spouses or adult children (Garcia-Ptacek et al., 2019) who provide unpaid care and support with activities of daily living (Happ et al., 2024). Caregivers of people with dementia commonly report high levels of caregiver burden, low quality of life, and depression and anxiety symptoms (Collins & Kishita, 2020; Sheehan et al., 2021; Wulff et al., 2020). With an increasing reliance on caregivers, support is needed to reduce caregiver burden, enhance well-being, and improve caregivers’ ability and willingness to provide care (Collins & Kishita, 2020; Zarzycki & Morrison, 2021).

### Background and Rationale

In Sweden, the national strategy for caregivers (Socialdepartementet, 2022) stipulates social care services should provide formal support to caregivers, including emotional, informational, and practical support (Johansson et al., 2021; Socialstyrelsen, 2017). Lethin, Leino-Kilpi, et al.’s (2016) mapping of formal caregiver support in Sweden found that support is provided either directly to the caregiver (e.g., caregiver education and psychological support), or indirectly (e.g., social care services provided to the person with dementia, such as respite care, that is, day care and home care services). Informal support, provided by family and friends, may also provide support of a similar value and function to formal support for caregivers (i.e., emotional, informational, and practical support; Koetsenruijter et al., 2015; Lethin et al., 2019).

Despite formal support service availability, caregivers in Sweden and internationally experience unmet needs, including a lack of informational (Lethin et al., 2019) and psychological support (Mansfield et al., 2022) and respite care (Lethin, Hallberg, et al., 2016). Lack of support is particularly pronounced for adult-child caregivers who report increased direct and indirect support needs compared to other caregiver populations (Cova et al., 2018; Kjällman-Alm et al., 2013). Research outside the Swedish context also suggests adult-daughter caregivers (daughter caregivers) experience more depressive symptoms than other caregiver populations (Martín-María et al., 2022; Watson et al., 2019). Daughter caregivers, who often take on primary caregiver responsibility, may face unique challenges, such as balancing care for parents and young children (Smith & Rodham, 2022), and combining work with their caregiving role (Vicente et al., 2022). Further, daughter caregivers may experience feelings of guilt toward their parent(s), leading to reduced engagement in leisure activities and an increased risk of depressive symptoms (Romero-Moreno et al., 2014). In the Swedish context there is a lack of research on the support experiences and needs of daughter caregivers, with research mainly focusing on partner/spousal caregivers (Johansson et al., 2021, 2024; Kjällman-Alm et al., 2013). Consequently, existing support may not be tailored to the needs and difficulties experienced by daughter caregivers.

Understanding daughter caregivers’ experiences of accessing and receiving support is important for developing an understanding of the context in which they provide care (Nilsen & Bernhardsson, 2019). Context is a multi-faceted concept and in complex interventions research typically refers to the setting in which an intervention may be implemented and includes cultural, economic, health system, organization, or social features (Skivington et al., 2021). One dimension of context is existing systems of support (Coumoundouros et al., 2024). Developing an understanding of context, alongside identifying potentially unmet support needs, are important first steps in developing complex healthcare interventions (O’Cathain et al., 2019; Skivington et al., 2021). The overall goal of the present study was to explore an important dimension of context that is, daughter caregivers’ existing formal and informal support systems. We aimed to explore the experiences of accessing and receiving formal and informal support among daughter caregivers of people living with dementia in Sweden.

## Methodology

### Qualitative Approach and Research Paradigm

We conducted a qualitative study using semi-structured interviews exploring the lived experience of daughter caregivers. We adopted a pragmatic research paradigm, selecting the best methods to address study aims and taking into consideration the potential transferability of results to other settings (Allemang et al., 2022; Morgan, 2007). Results are reported following Reflexive Thematic Analysis Reporting Guideline (RTARG; Appendix 1; Braun & Clarke, 2024).

### Researcher Characteristics and Reflexivity

We in the research team identify as men, women, and one as a queer woman. We have varied experiences of caregiving and living with chronic health conditions. Some have experience of both living with and being a caregiver of a close family member with a chronic condition, living with a chronic condition, being a caregiver of a parent with a neurodegenerative disorder, and experience of dementia in the wider family, with others having no experience of caregiving or living with chronic conditions.

Recruitment and data collection were conducted by a Swedish female doctoral student (Medical Science) and a Swedish female project coordinator (MSc Public Health). Data analysis was conducted by co-author OB, a Swedish doctoral student (Medical Science), with an MSc in Public Health and qualitative research training. OB has professional experience in conducting research related to people with dementia and caregivers’ needs in relation to psychological support. Data analysis supervision and theme development discussions were held with ACÅ, a Swedish professor (Medical Science), with both clinical and research experience, focusing on geriatrics, physical activity, cognitive impairment, and implementation science. She has extensive experience in qualitative research. Peer examination and supervision were provided by PF and JW. PF is an Australian professor (Evidence-Based Psychological Practice) residing in England with extensive experience in qualitative research. JW is a British associate professor (Caring Sciences) residing in Sweden and the principal investigator, with extensive experience conducting qualitative research, and her main area of research is informal caregiving.

Additional research team members read and commented on later versions of the thematic descriptions. Research team members included: AS, a research assistant with a MSc in Community and Public Health; RS, an associate professor in Gender Studies with extensive experience in qualitative research and research about older adult care and medical humanities; FS, a doctoral student in Medical Science, focusing on implementation of complex interventions in people with dementia; AB, an assistant professor with a focus on development and implementation of healthcare innovations; LvE, a professor in Caring Sciences and psychologist, focusing on mental health in people affected by somatic disease, with considerable experience in developing interventions for various caregiver populations. There were no pre-existing relationships between research team members and participants.

### Ethical Considerations

We obtained ethical approval from the Swedish Ethical Review Authority (Dnr 2020-05003, and Dnr 2021-00987) and written informed consent from all daughter caregivers. All procedures were in accordance with the Declaration of Helsinki.

### Participants

Eligible daughter caregivers were women aged 18 and over, able to speak, understand, and write in Swedish or English, and providing informal care for at least one community-dwelling parent (biological, non-biological, or parents-in-law) with dementia who was 65 years or older.

We used purposive sampling aiming to achieve maximum heterogeneity, that is, a demographically diverse sample of daughter caregivers (Fassinger, 2005). We recruited daughter caregivers across Sweden using a variety of recruitment strategies. These included leaflets and posters written in Swedish and English displayed in healthcare centers (i.e., primary care centers) in various locations, including ethnically and religiously diverse areas. Social media advertisements were placed in communities and organizations where daughter caregivers might be active (e.g., Demenscentrum, Anhörigas Riksförbund, and Facebook groups), alongside national and local caregiver communities and organizations, and groups for underserved populations (e.g., different religions, LGBTQ+, and women with immigrant backgrounds). This approach was adopted given social media tends to be biased towards white, young, middle-class, and more educated populations (Topolovec-Vranic & Natarajan, 2016). Daughter caregivers were also recruited via key persons from health and social care, and members of caregiver communities.

Interested daughter caregivers responded to study advertisements by contacting a research team member via telephone or email to receive a recruitment pack via the post or via the study website on the U-CARE Portal (Portal; https://www.u-care.se/; Sjöström et al., 2014, 2024). The Portal supports the digital execution of research studies, including online consent and data collection. The recruitment pack and study website included full study information, a consent form, and an eligibility questionnaire.

Daughter caregivers could provide informed consent either online via the Portal or by returning the consent form via the post using a stamped, addressed envelope included in the recruitment pack. The eligibility questionnaire was completed either online via the Portal, over the telephone, or via videoconference on the Portal with a research team member, or completed on paper and returned via the post.

Eligible daughter caregivers completed a background questionnaire online via the Portal, or via telephone or videoconference with a research team member. The background questionnaire collected data on demographics and screened for depressive symptoms using the depression subscale (DASS-D) of the Swedish translation of DASS-42 (Depression Anxiety Stress Scales; Eek, nd; Lovibond & Lovibond, 1995). Daughter caregivers were subsequently contacted by a research team member via telephone or email to arrange the interview.

### Participant Characteristics

Twenty-three daughter caregivers were recruited and interviewed. Daughter caregivers had a mean age of 51 years (SD = 9), the majority were Swedish (*n* = 21), with two having other European backgrounds. The majority had a university education (*n* = 16), held full-time employment (*n* = 15), and self-reported having a financially stable situation (*n* = 14). All were providing care to at least one biological parent with dementia, with 14 caring for one parent and nine also providing care to additional family members (e.g., the other parent, parent-in-law, or children). Daughter caregivers had been in a caring role for a mean of 8 years (SD = 7), and the majority did not reside in the same house as the parent (*n* = 22). To facilitate interpretation of supporting quotations, selected individual characteristics are provided in Table 1.

**Table 1.** Individual Characteristics of Daughter Caregivers

Pseudonym	Years caregiving	Age category	Caring hours per week	Caring for more than parent with dementia	Employment	Depression Severity (DASS-D)^a^
Astrid	30–39	50–59	10–19	No	Full-time	Moderate
Carolina	10–19	60–69	Missing	Yes	Jobseeker	Normal
Christa	10–19	50–59	10–19	No	Full-time	Extremely severe
Cleo	10–19	50–59	0–9	Yes	Full-time	Mild
Ebba	10–19	50–59	Missing	Yes	Full-time	Moderate
Elvira^b^	10–19	40–49	20–29	Yes	Full-time	Mild
Esther	10–19	40–49	0–9	No	Full-time	Normal
Gerd	0–9	60–69	10–19	No	Retired	Moderate
Hildur	0–9	60–69	0–9	No	Full-time	Normal
Iris	0–9	60–69	0–9	No	Sick leave	Moderate
Irma	0–9	50–59	10–19	Yes	Part-time	Normal
Julia	0–9	50–59	10–19	No	Full-time	Severe
Leia	0–9	50–59	10–19	No	Jobseeker/Voluntary work	Normal
Lotta	0–9	50–59	0–9	No	Full-time	Normal
Marianne	0–9	50–59	0–9	No	Student	Severe
Molly	0–9	50–59	Missing	Yes	Full-time	Moderate
Monika	0–9	40–49	30–39	Yes	Full-time	Normal
Nora	0–9	40–49	10–19	Yes	Part-time/Sickness benefit	Normal
Selma	0–9	40–49	0–9	Yes	Full-time/Student	Moderate
Sigrid	0–9	40–49	0–9	No	Full-time	Moderate
Stella	0–9	40–49	0–9	No	Full-time	Moderate
Vera	0–9	30–39	10–19	No	Part-time/Business owner	Normal
Wilma	0–9	30–39	0–9	No	Full-time	Normal

*Note.* Pseudonyms assigned and ages provided in bandings to protect confidentiality.

^a^DASS-D (Depression Anxiety Stress Scale - Depression sub-scale) cut-offs: Normal 0–9 (i.e., no depression); Mild 10-13; Moderate 14-20; Severe 21-27; Extremely severe 28+ (Lovibond & Lovibond, 1995).

^b^Living together with parent.

### Dataset Generation

We conducted semi-structured interviews to explore daughter caregivers’ experiences in-depth between September 2021 and May 2022. Interviews ranged from 32 min to 2 hr 49 min, with a mean duration 1 hr 24 min. We conducted interviews face-to-face (*n* = 4), via the telephone (*n* = 12), or videoconference (*n* = 6) via the Portal, and one interview started as videoconference and changed to telephone due to technical issues. Interviews followed a topic guide, with subsequent questions open and flexible to responses to capture both our topics of interest, daughter caregivers’ own views, and any unanticipated findings (Britten, 1995). A topic guide, partially informed by previous research (Woodford et al., 2018), explored (a) experiences of seeking support, (b) experiences of receiving support, and (c) preferences for psychological support, with additional questions for caregivers with elevated depressive symptoms (DASS-D score ≥14; Appendix 2).

Following a reflexive thematic analysis approach (Braun & Clarke, 2022), we did not adhere to the concept of data saturation (Saunders et al., 2018). Instead, daughter caregivers were recruited and interviewed in-depth until we deemed that we had collected enough data with sufficient information power to develop rich and meaningful themes (Braun & Clarke, 2019; Malterud et al., 2016).

We made audio recordings of interviews. We assigned each caregiver a unique identification number and a pseudonym name, and stored audio recordings on a password-protected shared drive on a secure server along with the identification number. A professional transcription company transcribed interviews verbatim, and we uploaded transcripts to NVivo V.12.0 to support analysis. Transcribed data was de-identified in terms of names, places, and other identifiable characteristics of the caregivers. Analysis started after data collection was completed.

### Data Analysis

We adopted a six-step reflexive thematic analysis approach, which is well-suited to explore daughter caregivers’ lived experience due to its flexibility and emphasis on researcher reflexivity (Braun & Clarke, 2022). We used an inductive approach primarily focused on semantic meaning for coding and theme development. OB conducted the data analysis, and started by reading interview transcripts to familiarize with their overall structure and content. OB wrote initial annotations for data potentially meaningful for the research aim, and subsequently coded all data relevant to the aim. Subsequently, all codes were collated into preliminary themes and subthemes, which were iteratively reviewed and refined (Braun & Clarke, 2022). OB developed thematic maps and theme descriptions to illustrate and aid interpretation of themes and subthemes (Braun & Clarke, 2022). Several iterations of thematic maps were generated to illustrate how the analysis process developed (Appendix 3). Throughout the process, OB received iterative supervision, discussion, and peer examination of the analysis with senior research team members (PF, JW, and ACÅ), allowing for ongoing refinement of impressions and reflections on the data. Transparency was maintained by keeping an audit trail with reflexive journaling and memos (Williams & Morrow, 2009). Supporting quotations were translated by native Swedish and native English-speaking research team members. Quotations have been edited to clarify language by removing hesitations and repetitions, which have been replaced with an ellipsis (“…”). Clarifications have been added in brackets in quotations.

## Findings

We generated two themes, Types of support and Mismatched support. The first theme is context-related and describes the different types of formal and informal support daughter caregivers expressed receiving. The second theme highlights ways in which support was considered inadequate to meet daughter caregivers’ needs, that is, an experienced mismatch between accessed and received support and support needs. Within each theme, specific subthemes were generated (Table 2). Whilst the first theme, Types of Support, could be considered a topic summary (Braun & Clarke, 2023), this theme and its subthemes help build the narrative leading to the second theme, Mismatched support.

**Table 2.** Thematic Structure

Theme	Summary	Subthemes
Types of support	Daughter caregivers described receiving both direct and indirect formal or informal support. Direct support was primarily aimed at supporting the daughter caregivers, including *Sharing experiences* (e.g., peer support groups and online forums with other caregivers), and *Professional support* (e.g., psychological support, and social support services providing individual caregiver support). Indirect support (e.g., respite care services including day care and home care) was primarily aimed at supporting the person with dementia, but alleviated the daughter caregivers from their caregiving duties, resulting in *Getting some relief.*	Sharing experiences
		Professional support
		Getting some relief
Mismatched support	Daughter caregivers perceived some types of accessed support as generally inadequate with a *Lack of tailoring to daughter caregivers’ need*s. Disorganized and fragmented formal support resulted in *Difficulties navigating support*, negatively impacting daughter caregivers’ opportunities to access support.	Lack of tailoring to daughter caregivers’ needs
		Difficulties navigating support

### Theme 1: Types of Support

Subthemes *Sharing experiences, Professional support,* and *Getting some relief* further explore daughter caregivers’ experiences of receiving different types of support.

#### Sharing Experiences

For most daughter caregivers, receiving peer support and *Sharing experiences* was described as providing a sense of *“unity, to know others in the same situation”* (Gerd), reducing feelings of isolation, and helping to normalize their situation.

“There is a huge difference in meeting others in the same situation than meeting... your friends actually. You need to have this implicit understanding that is just there, that you cannot even say.” (Monika)

Family and friends were described as providing this type of support, as expressed by Iris: *“I have my sister, so we vent a lot”*. However, as Sigrid described, gaining a shared understanding of the caregiving experience with others was hard *“unless you are in it yourself”.*

Daughter caregivers described a general need to talk to others: *“People have such a need to talk about their situation or how difficult it is… I think you kind of need to talk”* (Vera). Group support (e.g., social care services providing peer support groups, or caregiver cafés) provided caregivers with this opportunity. For most, normalization of the caregiving situation was perceived as the most important benefit of *Sharing experiences*. Group support was further highlighted as alleviating loneliness in the caregiving situation: *“It is just that it* [group support] *is very valuable, that there are more people who feel like me, that there are many people who have it… more or less like me”* (Christa). Some daughter caregivers described online forums as bringing similar benefits as group support.

“I am in this dementia forum on Facebook, which is a fantastic place. It is an incredibly humble tone and a lot of people share their experiences about their parents... everyone is in the same situation.” (Nora)

Using group support and online forums was also described to get *“tips and advice”* (Iris) on how to manage their situation from others. Additional ways of finding information included listening to podcasts and searching for information on the internet, with Stella explaining: *“I have spent so much time learning things* [about dementia] *from Facebook groups and Googling things”*. However, Elvira, who did not have any forum to meet other caregivers, expressed: *“I never get to hear stories from others who care for a person with dementia… or just this confirmation that I am not alone”*. In response to not being offered group support, Carolina described “*I started my own little network *[of caregivers]”, further highlighting the need for caregivers to share experiences.

#### Professional Support

Daughter caregivers with experience of *Professional support*, perceived it to help empower them in their caregiving role, manage their emotions, and carry out practical caregiving tasks. Receiving psychological support, such as talking therapy, was described as important. Daughter caregivers expressed a need to talk about their caregiving situation with a professional, with some describing discussing different solutions with professionals to address caregiving difficulties as helpful to manage their mood and situation. Some also expressed psychological support as facilitating their ability to step out of their caregiving situation, gain perspective, and feel empowered in the caregiving role. Marianne shared: *“When you have talked out loud about the situation, then all of a sudden it feels like it was not that bad”*. Further, some described psychological support as helping them to take control of their situation by providing tools on how to manage thoughts and emotions.

“It [psychological support] is probably the best thing, getting tools and being able to train myself to use them. It [psychological support] then helped me to gain strength, a way to relate to myself” (Cleo)

However, for some, the positive benefits of psychological support were perceived as dependent on the relationship with the support provider, with Elvira expressing that *“not one size fits all”*. Some further described how a poor relationship with the person providing psychological support could be disempowering and voiced a need to: *“Find someone you feel you can work with, that you can be sincere with, that you trust”* (Christa).

Most daughter caregivers highlighted that receiving information about dementia and informal caregiving empowered them in their caregiving role. For example, Wilma described caregiver support services as being an important source for *“practical information about the different support options available”*. Practical information was provided by caregiver support services alongside contact information about organizations providing different types of caregiver support. Julia explained: “*I have talked to them* [caregiver support] *a few times when I did not know what to do* [to receive support]*”*, describing caregiver support services as helpful and highlighting the importance of professional support to empower caregivers.

#### Getting some Relief

Daughter caregivers described *Getting some relief* when social care was provided to their parent with dementia, for example, day care or home care, alleviating them from caregiving duties. Home care services alleviated them from practical duties, allowing time to focus on supporting their parent in maintaining their selfhood by engaging in meaningful activities with them.

“The home care can cook and clean, and we can go for a walk with Dad and talk about memories, the home care cannot.” (Leia)

Similar to respite care services, most described *Getting some relief* when other family members carried out caregiving duties, easing the burden of care. However, those without family members or friends were fully reliant on formal respite care services, with Sigrid explaining: *“we have received day care and even more help at home* [from home care]*, I feel like it would never have been possible without* [this support]*”*.

Whilst home care services were described by some as being helpful, and important by daughter caregivers, others described it as poor, not being on time, and *“really strange”* (Cleo), which resulted in increased burden instead of providing relief. For example, changes in home care staff were described by some as causing worry and impacting the quality of care provided to their parent. Additionally, some home care service staff were perceived as lacking appropriate training to provide care for people with dementia, and being overly reliant on daughter caregivers for information.

### Theme 2: Mismatched Support

Subthemes *Lack of tailoring to daughter caregivers’ needs,* and *Difficulties navigating support* further explore daughter caregivers’ experiences of accessed support.

#### Lack of Tailoring to Daughter Caregivers’ Needs

Caregiver support was perceived as poorly adapted to the situation and needs of daughter caregivers. Some daughter caregivers perceived an expectation for them to manage caregiving duties alone. Additionally, support was considered more commonly tailored to partner/spousal caregivers living together with the person with dementia.

“If you are the spouse of someone, then you can get support and relief, but there is no such thing for adult children. Home care is their support and relief, which is directly aimed at my father… I suggest support was introduced for the [adult] child [caregiver] as well.” (Marianne)

Daughter caregivers emphasized that support should be tailored to match the needs of both the caregiver and the person with dementia, especially as Carolina expressed, both will *“need more and more help”* as symptoms progress. Following a diagnosis of dementia, the lack of support tailored to their needs was highlighted, as they stepped into the role of caregiver to their parent. Sigrid described receiving no support after diagnosis: *“*[I wished] *that someone had picked us up already when we* [parent with dementia] *were diagnosed and not just sent us home”*. Further, most expressed frustration that the timing of support did not match the level of dementia progression and subsequent increasing needs for caregiver support: *“In summary, no one is taking the initiative to push it* [caregiver support] *forward”* (Stella). Caregiver support was also described as coming too late, with no one coordinating and leading support efforts.

“I had thought that we had come further, there are so many people today who have dementia and who have illnesses, and there are so many informal caregivers who help their relatives, that there should be understanding in health and social care and… I have not found that.” (Carolina)

Lack of support was perceived by some as being partly explained by a lack of understanding from health and social care about the support needs of daughter caregivers.

#### Difficulties Navigating Support

Daughter caregivers described *Difficulties navigating support* due to perceptions that available support was disorganized and fragmented. Navigating support was described as taking up a lot of time and energy, despite already being highly burdened. To both manage care for their parent and receive support for themselves, daughter caregivers needed to stay in contact with several different care providers. Monika described how these care providers failed to communicate with each other.

“It took a while to understand the whole circus with everything [caregiver support]. What help is there, who to call? What is the difference between a municipality and a county council [region]? You feel quite small and to get all the different care providers in order, who does what [care provision].”

Stella also highlighted: *“Frustration around finding and navigating the support efforts that exist”*, adding to caregiver burden. Molly described her experiences of discovering caregiver support through informal sources as opposed to formal care pathways: *“I think it was through a neighbor, I heard about something called caregiver support, which the municipality provides”*. Most expressed experiencing a lack of understanding about how to navigate caregiver support services, resulting in not feeling in control of their situation.

“There is no common thread, red thread, you are just tossed around … There is really no support and no information for caregivers. As a caregiver you are completely at the mercy of others.” (Ebba)

There was one disconfirming case, whereby Iris did not mention *Difficulties navigating support*, as she described receiving help from a care navigator.

“[We have] a care navigator who helps us with contact with the municipality [social care], county council [region healthcare] and everything. Otherwise, there will be a lot of calling around and keeping track of everything. From what I have understood, it is not all county councils [regions] that have that [support].”

As Iris expressed, there are solutions to navigating support; however, as available caregiver support varies and is dependent on what is offered in the municipality or region in Sweden where caregivers live, it is not accessible to most daughter caregivers.

## Discussion

To our knowledge, this is the first study to explore the contextual dimension of caregivers’ existing systems of formal and informal support in Sweden with a specific focus on the experiences of daughter caregivers of at least one parent with dementia. Our findings extend previous research on dementia caregivers by describing the context of caregiver support in Sweden and presenting the experiences of daughter caregivers who perceive a mismatch between the formal support received and their specific support needs. Types of support received included peer support, providing an opportunity for *Sharing experiences*, reducing feelings of isolation, and normalizing the caregiving situation, and *Professional support* was found to empower and help daughters manage the caregiving role. Daughter caregivers experienced *Getting some relief* from caregiving duties via indirect formal support for the person with dementia. Despite receiving support, daughter caregivers experienced Mismatched support, lacking adaptation to their needs. This was identified as *Lack of tailoring to daughter caregivers’ needs*, where formal support was not adapted to the specific needs experienced, nor tailored to increasing needs as dementia progresses. Daughter caregivers also experienced *Difficulties navigating support*, due to the complex structure of caregiver support, limiting opportunities to access support.

As reported in our findings, various types of formal support were described as being available in Sweden, including group support (such as peer support), psychological support, informational support, and respite care (Lethin, Leino-Kilpi, et al., 2016; Vandepitte et al., 2016a, 2016b). Formal support has been found to reduce caregiver burden, improve mental health and well-being, enhance quality of life, and ensure better care for the person with dementia (Antelo Ameijeiras & Espinosa, 2023; Jackson & Browne, 2017). However, research also shows that although caregiver support is offered throughout the dementia disease trajectory in some countries, including Sweden, access remains low (Lethin, Leino-Kilpi, et al., 2016). According to our findings, a main factor perceived by daughter caregivers as contributing to the underutilization of caregiver support is the mismatch between available formal support and caregivers’ specific needs.

The mismatch between available formal support and daughter caregivers’ specific needs contrasts with the Swedish strategy for caregiver support, which recommends providing individualized and flexible support (Socialdepartementet, 2022). The need for individualized and flexible support adapted to caregivers’ needs has been identified in Australian and Swedish studies within a general caregiver population of people with dementia (Lethin et al., 2018; Tatangelo et al., 2018). Our study extends these findings, with daughter caregivers reporting specific challenges related to the lack of tailored support, such as not living with a parent with dementia, affecting their access to, for example, respite care services. Caregiver support services that fail to provide support according to needs, that is, offering mismatched support, may have a reduced ability to effectively support that caregiver population (Morrisby et al., 2021).

Another dimension of *Lack of tailoring to daughter caregivers’ needs* concerns the timeliness of support and the need to adapt support to address increasing caregiver burden as dementia progresses (Kokorelias et al., 2022; Lethin, Leino-Kilpi, et al., 2016; van den Kieboom et al., 2020). In the early dementia stages, research has found caregivers express unmet needs concerning dementia information and the caregiving role, leading them to feel ill-equipped post-diagnosis to undertake the caregiving role (Bressan et al., 2020; Laparidou et al., 2019; Lethin, Hallberg, et al., 2016). Our findings further highlight increasing support needs for both the caregiver and person with dementia as dementia symptoms progress. Barriers to timely home care support as dementia progresses include inconsistent support delivery, poor coordination between services, lack of outreach, and not receiving information about support services (Dalgarno et al., 2021). Similar findings have been found with daughter caregivers in the United Kingdom (Smith & Rodham, 2022) and a general caregiver population of people with dementia in Europe (Jelley et al., 2021). Daughter caregivers’ need for information about dementia and available services has been highlighted elsewhere (Dang et al., 2022; Soong et al., 2020).

Previous research has found different caregiver groups (e.g., spousal, adult child, and adult sibling) to have both common and unique needs (Dang et al., 2022). Needs such as information about the care recipient’s health condition and service availability (e.g., respite services) were found to be common needs. However, adult child caregivers report more challenges with poor care coordination than spousal caregivers (Dang et al., 2022). Daughter caregivers in our study highlighted *Difficulties navigating support*, with available health and social care support for caregivers and people with dementia described as having a complex structure. Australian daughter caregivers report similar challenges navigating support (Morrisby et al., 2021; Poisson et al., 2023). Daughter caregivers found navigating support frustrating, perceiving support services as failing to reach out to them. Daughter caregivers reported similar challenges in the United Kingdom (Smith & Rodham, 2022) and adult child caregivers have expectations for better coordination from health and social care professionals than spousal caregivers (Peeters et al., 2010). Poor care coordination may be particularly challenging for daughter caregivers, as they may be more likely to balance care for parents and young children (Smith & Rodham, 2022) and combine work with their caregiving role (Vicente et al., 2022). Lack of outreach, alongside fragmented and uncoordinated support identified in the present study, have been found to limit access to formal support in Sweden (Lethin et al., 2018). Poor communication between care providers and insufficient information on how to navigate available support services can contribute to a lack of timely and tailored support provision for caregivers (Laparidou et al., 2019). The development of care coordination interventions to address challenges with disorganized and fragmented care may be particularly important for daughter caregivers.

In regards to informal support, our findings highlighted the importance of peer support with respect to *Sharing experiences* with peers. This involved *Sharing experiences* to help others, as well as learning from those facing similar situations (Eronen, 2020). *Sharing experiences* helped alleviate feelings of loneliness, in line with research in a general caregiver population (Lauritzen et al., 2019). Furthermore, in line with previous literature, peer support was found to help caregivers manage challenges (Smith et al., 2018) and improve their daily lives, while also improving their mental health (Halvorsrud et al., 2020). Online forums were described in our findings as another way of *Sharing experiences.* Other research has found *Sharing experiences* in online forums can provide feelings of competence and social value (McKechnie et al., 2014). Additionally, whilst the majority of online form users often participate by “lurking”, that is, reading about others’ experiences rather than posting themselves (Skog et al., 2023), benefits are similar to interacting in the forum (McKechnie et al., 2014). Online support may represent a way to increase access to peer support for caregivers (Du et al., 2021), especially for caregivers at a distance or those without respite services (McLoughlin et al., 2023), and may also reduce healthcare costs (Carter et al., 2020).

However, access to informal support was dependent on daughter caregivers’ existing social network (i.e., family and friends) and the willingness and ability of this network to provide support. Some daughter caregivers in the present study lacked supportive social networks, turning to online forums to form new social networks (Davies et al., 2019). While such online social networks can provide emotional and informational support, they do not provide relief from practical caregiving duties. Daughter caregivers lacking social networks may therefore be more dependent on the provision of formal support services, for example, home care and respite care. However, given some daughter caregivers perceived such services to be tailored towards partner/spousal caregivers, this further underscores the need to ensure such services are accessible and tailored to different caregiving groups.

Interestingly, our findings suggest daughter caregivers experience a mismatch between available support and their needs, specifically regarding formal support services rather than informal support. This finding contrasts with previous research on spousal caregivers, which found a mismatch between the supply and demand of social support (Dam et al., 2018). Daughter caregivers often experienced formal support that was poorly aligned with their specific caregiver context, e.g., support was perceived as generic or more commonly tailored to partner/spousal caregivers. In contrast, support from family, friends, and peers (where available) was described as helpful in reducing isolation and normalizing their situation. This finding suggests that the provision of peer support may represent a way to tailor support to the needs of daughter caregivers, that is, by providing more context-specific and relevant emotional and instrumental support.

### Clinical Implications

Currently, daughter caregivers in Sweden receive various types of formal and informal support. However, formal support is not perceived as adapted to their unique needs. Existing caregiver support and previous research are predominantly focused on partner/spousal caregivers, who have different needs compared to daughter caregivers. This disparity may reduce access to support for daughter caregivers (Dang et al., 2022; Johansson et al., 2021). Our findings suggest a need to develop interventions tailored to the needs of daughter caregivers and adapted to different stages across the dementia trajectory. This is supported by previous research in Sweden (Lethin, Hallberg, et al., 2016) and other contexts (Huertas-Domingo et al., 2023; Smith & Rodham, 2022). Further, given caregivers experience a wide range of support needs (e.g., related to finance, physical health, and social well-being), interventions need to go beyond only psychological support and reducing caregiver burden (Spiers et al., 2024).

Adopting a collaborative care model within a primary care framework may represent a potential solution to improve collaboration between formal health and social care services (Mavandadi et al., 2017). In a collaborative care model, case managers, caregivers, and health and social care providers are involved in care. Trained case managers routinely assess the needs of people with dementia and caregivers and provide access to evidence based interventions dependent on needs of the person with dementia (i.e., delusions or hallucinations, depression, personal care, mobility, sleep), and the needs of caregivers (i.e., anxiety, depression, stress; Mavandadi et al., 2017). Case managers can also facilitate access to appropriate health and social care services. Benefits of collaborative care models include a reduction in caregiver burden and psychological distress, and reduced nursing home placement of people with dementia (Mavandadi et al., 2017). An alternative model to overcome difficulties navigating complex health and social care systems is dementia care navigation, whereby non-clinical professionals (e.g., care navigators) are in regular contact with people with dementia and caregivers to signpost to suitable local health and social care and support services (Giebel et al., 2023).

Peer support groups and online forums may offer an accessible solution to address some of the unmet support needs of daughter caregivers (Du et al., 2021; Lauritzen et al., 2019; McKechnie et al., 2014), such as the provision of emotional support and information (Yin et al., 2024). However, peer support interventions tailored to specific caregiver groups of people with dementia need to be developed and evaluated (Carter et al., 2020). Experienced current or former caregivers can provide volunteer peer support (Smith et al., 2018) or peer support can be provided formally through formally trained peer support workers (Fortuna et al., 2019). Countries such as the United Kingdom have developed competency frameworks for peer support workers (Roth & Pilling, 2020) and such workers are being introduced into mental health care across Sweden (Rosenberg & Argentzell, 2018). Using non-clinical professionals, for example, peer support workers can be equally effective as clinical professionals and could increase access to support for caregivers (Lidbetter et al., 2024). As a next step, peer support could be explored as a possible component of caregiver support in Sweden.

### Limitations

As caregiver support is provided by 290 municipalities in Sweden, there is heterogeneity in the support provided throughout the country depending on resources, demographics, and priorities (Meinow et al., 2022). Whilst daughter caregivers were recruited across Sweden, findings may not be representative of each municipality. It is possible caregivers who chose to participate did so because they perceived a lack of support, which may have led to the predominance of negative support experiences reported. Despite using a variety of recruitment strategies aiming to recruit underserved and minority populations, the sample was homogeneous, mainly consisting of middle-aged, well-educated, and middle-class Swedish women. Social media can be a suitable method to recruit underserved populations; however, it has also been shown to have a bias toward white, young, and educated women (Topolovec-Vranic & Natarajan, 2016). Further, only Swedish or English-speaking daughter caregivers were eligible, meaning speakers of minority languages who are not fluent in Swedish or English were excluded. Future research should explore the specific needs of minority daughter caregiver populations to ensure the development of equity-focused support (Chejor et al., 2022). Public contributors were not involved in this study and their contribution could have addressed some limitations discussed (e.g., developing recruitment strategies, co-designing of the topic guide, and contributing to data analysis; Brett et al., 2014).

Despite the aforementioned limitations, this study has several strengths. To ensure trustworthiness, discussions and peer examination were conducted with senior research team members with extensive experience in qualitative caregiving research (Williams & Morrow, 2009). Furthermore, we adopted an iterative data analysis procedure, and as themes and subthemes were generated, they were repeatedly checked and confirmed against interview data (Braun & Clarke, 2022).

### Conclusions

Daughter caregivers received formal and informal support from a variety of services. However, a mismatch was experienced between the type of support accessed and received and specific support needs. To overcome this challenge, developing support better tailored to the needs and situation of daughter caregivers of people living with dementia in Sweden is needed. Developing an understanding of this important dimension of context, that is, daughter caregivers’ existing formal and informal support systems, represents an important first step in the development of interventions for daughter caregivers better tailored to their unique needs (O’Cathain et al., 2019). Specifically, the potential of exploring collaborative care models, dementia care navigation, and utilizing different types of peer support warrants further research.

## Supplemental Material

Supplemental Material - Experiences of Formal and Informal Support Among Adult-Daughter Caregivers of People With Dementia in Sweden: A Qualitative Study Supplemental Material for Experiences of Formal and Informal Support Among Adult-Daughter Caregivers of People With Dementia in Sweden: A Qualitative Study by Oscar Blomberg, Abla Sami, Paul Farrand, Renita Sörensdotter, Frida Svedin, Anders Brantnell, Louise von Essen, Anna Cristina Åberg, and Joanne Woodford in Dementia

## Data Availability

The datasets generated and/or analyzed during the current study are not publicly available due to privacy or ethical restrictions but are available from the corresponding author on reasonable request.*
